# Trans fatty acids in adipose tissue and risk of myocardial infarction: A case-cohort study

**DOI:** 10.1371/journal.pone.0202363

**Published:** 2018-08-22

**Authors:** Marianne Uhre Jakobsen, Anders Gorst-Rasmussen, Helle H. Eriksen, Jakob Stegger, Albert M. Joensen, Anne Tjønneland, Jørn Dyerberg, Erik B. Schmidt, Kim Overvad

**Affiliations:** 1 Section for Epidemiology, Department of Public Health, Aarhus University, Aarhus, Denmark; 2 Division for Diet, Disease Prevention and Toxicology, National Food Institute, Technical University of Denmark, Kgs. Lyngby, Denmark; 3 Department of Cardiology, Aalborg University Hospital, Aalborg, Denmark; 4 Unit of Epidemiology and Biostatistics, Aalborg University Hospital, Aalborg, Denmark; 5 Departments of Anaesthesiology and Intensive Care Medicine and Clinical Medicine, Aalborg University Hospital, Aalborg, Denmark; 6 The Danish Cancer Society Research Center, Copenhagen, Denmark; 7 Department of Nutrition, Exercise and Sports, University of Copenhagen, Frederiksberg, Denmark; University of Illinois, UNITED STATES

## Abstract

**Background:**

The risk of coronary heart disease associated with intake of individual trans fatty acids (TFAs) is not clear. Adipose tissue content of TFAs is a biomarker of TFA intake and metabolism.

**Objective:**

We investigated the rate of myocardial infarction (MI) associated with the adipose tissue content of total 18:1t, isomers of 18:1t (18:1 Δ6-10t and 18:1 Δ11t) and 18:2 Δ9c, 11t.

**Methods:**

A case-cohort study, nested within the Danish Diet, Cancer and Health cohort (n = 57,053), was conducted, which included a random sample (n = 3156) of the total cohort and all incident MI cases (n = 2148) during follow-up (14 years). Information on MI cases was obtained by linkage with nationwide registers and validated. Adipose tissue was taken from the participants buttocks and the fatty acid composition was determined by gas chromatography.

**Results:**

Women with higher adipose tissue content of total 18:1t had a 57% higher MI rate (quintiles 5 versus 1, hazard ratio, 1.57; 95% confidence interval, 1.12–2.20; P-trend = 0.011) and women with higher content of 18:1 Δ6-10t had a 76% higher MI rate (quintiles 5 versus 1, hazard ratio, 1.76; 95% confidence interval, 1.23–2.51; P-trend = 0.002). No association between 18:1 Δ11t content and MI rate was observed. In men, no associations between adipose tissue content of total 18:1t and 18:1 Δ6-10t and MI rate were observed. However, men with higher content of 18:1 Δ11t had a 48% higher MI rate (quintiles 5 versus 1, hazard ratio, 1.48; 95% confidence interval, 1.17–1.86; P-trend = 0.003). Adipose tissue content of 18:2 Δ9c, 11t was not associated with MI rate in women or men.

**Conclusions:**

Adipose tissue content of 18:2 Δ9c, 11t was not associated with MI rate in women or men, whereas higher contents of isomers of 18:1t were associated with higher MI rates but the associations for individual 18:1t isomers differed, however, in women and men.

## Introduction

Trans fatty acids (TFAs) are unsaturated fatty acids with at least one double bond in the trans configuration and formed either by biohydrogenation in ruminants or by industrial hydrogenation. The predominant products are isomers of 18:1t (*trans*-octadecenoic acid), but in different proportions [1,2]. Industrial, partially hydrogenated vegetable oils (i.e., margarines and shortenings) mainly contain the 18:1t isomers 18:1 Δ9t (*trans*-9-octadecenoic acid, elaidic acid) and 18:1 Δ10t (*trans*-10-octadecenoic acid), whereas 18:1 Δ11t (*trans*-11-octadecenoic acid, vaccenic acid) is the major 18:1t isomer in the meat and milk fat of ruminants. 18:1 Δ11t is also the major precursor of 18:2 Δ9c, 11t (*cis*-9, *trans*-11-octadecenoic acid, rumenic acid) belonging to the conjugated linoleic acid family [3].

Observational epidemiological studies have shown that a high habitual intake of TFAs from industrial sources is associated with a higher risk of coronary heart disease, whereas an association with intake of TFAs from ruminant sources has been questioned [4–9]. The conflicting findings in observational epidemiological studies could be explained by other nutrients in foods that contain TFAs, by dietary patterns underlying TFA intake or by a lower TFA intake from ruminant sources. Furthermore, different TFA subtypes (of varying chain length and unsaturation) and isomers (of similar chain length and unsaturation, but different location of double bonds) may have divergent effects [9]. However, the risk of coronary heart disease associated with intake of different 18-carbon TFA subtypes and isomers has only been investigated in a few studies, leaving the coronary heart disease risk associated with intake of 18-carbon TFA subtypes and isomers in general unaddressed.

Adipose tissue content of TFAs is a long-term biomarker of TFA intake and metabolism [10]. It follows that adipose tissue content of TFAs reflects endogenous exposure to TFAs and therefore presumably a more biologically relevant measure of exposure to TFAs than intake of TFAs. Moreover, adipose tissue content of TFAs is an objective measure minimizing random measurement error. Thus, adipose tissue content of TFAs offers an intriguing and relevant possibility to investigate the health effects of different TFA subtypes and isomers. Only a few studies have investigated the risk of coronary heart disease associated with the adipose tissue content of different 18-carbon TFA subtypes and isomers. In a population-based case-control study, a higher rate of myocardial infarction (MI) was found for a high content of 18:1 Δ11t, whereas no associations were found for the content of 18:1 Δ9t and 18:1 Δ10t [11]. In another population-based case-control study, a lower rate of MI was observed for a high content of adipose tissue 18:2 Δ9c, 11t [12].

We attempted to address this key gap in the existing literature by investigating the rate of MI associated with the percentage of total 18:1t (*trans*-octadecenoic acid), isomers of 18:1t (18:1 Δ6-10t and 18:1 Δ11t) and 18:2 Δ9c, 11t (*cis*-9, *trans*-11-octadecenoic acid) in adipose tissue in both women and men.

## Methods

### Study design and population

Between December 1993 and May 1997, 160,725 women and men living in the greater Copenhagen and Aarhus areas were invited to participate in the Danish Diet, Cancer and Health cohort [13]. The criteria for invitation were the following: age between 50 and 64 years, born in Denmark and no diagnosis of cancer registered in the Danish Cancer Registry. In total, 57,053 persons (35%) accepted the invitation and were enrolled. Prior to the visit to one of two study centers, all participants completed a 192-item semi-quantitative food-frequency questionnaire. At the visit to the study center, participants filled in an additional questionnaire on lifestyle and medical history. The self-administered questionnaires were checked by laboratory technicians who also performed anthropometrical measurements of the participants. Furthermore, an adipose tissue biopsy was taken from the buttock with the use of a Luer lock system (Terumo; Terumo Corp.), according to the method of Beynen & Katan [14]. The samples were flushed with nitrogen and stored at -80°C until analysis.

We conducted a case-cohort study [15], nested within the cohort, which included a subcohort (random sample of the total cohort) and all incident MI cases (S1 Fig). The Diet, Cancer and Health cohort has been approved by the Health Research Ethics, the Capital Region of Denmark and the Danish Data Protection Agency. Written consent including permission for prospective data collection from national registries was obtained from all participants prior to enrollment.

The data used in the current study will be made available upon request after application to the Diet, Cancer and Health Executive Committee. The application form can be obtained from Louise Hansen (louhan@cancer.dk).

### Analysis of adipose tissue content of TFAs

The fatty acid composition in adipose tissue was determined using gas chromatography as described in detail previously and the content of fatty acids was expressed as weight percentages of total fatty acids [16]. Commercially available standards (Nu-Chek-Prep, Inc.) were used to identify the individual fatty acids. The following TFAs were identified: 18:1 Δ6t (+Δ8t), 18:1 Δ9t, 18:1 Δ10t, 18:1 Δ11t and 18:2 Δ9c, 11t. The inter-assay coefficient of variations for the determination of the 18:1t isomers were between 3.7% and 7.1%, and the inter-assay coefficient of variation for the determination of 18:2 Δ9c, 11t was 2.9%. The pairwise correlations between the 18:1t isomers 18:1 Δ6t (+Δ8t), 18:1 Δ9t and 18:1 Δ10t were between 0.88 and 0.90. Consequently, we decided to merge these fatty acids into one exposure category labelled 18:1 Δ6-10t. The pairwise correlations between 18:1 Δ11t and the other 18:1t isomers were much lower (0.29–0.47). Total 18:1t was defined as the sum of 18:1 Δ6-10t and 18:1 Δ11t. Thus, the study exposures were adipose tissue content of total 18:1t, 18:1 Δ6-10t, 18:1 Δ11t and 18:2 Δ9c, 11t.

### Identification of MI cases

The study outcome was incident MI (fatal and non-fatal). Information on incident cases of MI was obtained by linkage with nationwide registers. Participants who were registered with a diagnosis of MI according to the International Classification of Diseases (ICD)-8 codes 410–410.99 and ICD-10 codes I21.0-I21.9 in the Danish National Patient Register or the Danish Register of Causes of Death were identified. Furthermore, participants who were registered with a diagnosis of cardiac arrest (ICD-8 codes 427.27 and ICD-10 codes I46.0–I46.9) were identified and included if the arrest was considered to be of cardiac origin after validation. A validation study, based on a complete review of all medical records from enrollment through 2003, showed that the positive predictive value for MI discharge diagnoses in the Danish National Patient Register was 92.4% among those who received an MI diagnosis in a hospital ward [17]. Thus, from January 2004, all participants registered with an MI diagnosis and discharged from a ward were accepted as cases without further validation. All other potential cases were individually validated [18]. Incident cases of MI were classified as fatal if death occurred within 28 days of hospital admission for an MI based on data from the Civil Registration System.

Participants were followed from enrollment into the Diet, Cancer and Health cohort until the first registration of MI, death from another cause, emigration or end of follow-up 31 December 2009, whichever came first. In supplementary pre-specified analyses, the study was halted 30 June 2001 after approximately five years of follow-up due to the presumed declining exposure to industrial TFAs during follow-up; after Denmark imposed a regulation specifying a maximum of industrially produced TFAs in oil or fat intended for human consumption.

### Covariates

Potential confounders of the association between the adipose tissue content of TFAs and the rate of MI were selected a priori, based on the existing literature on risk factors for coronary heart disease, and included as covariates in the analyses. Information on length of education, smoking, leisure time physical activity, hypertension and diabetes mellitus was obtained from the lifestyle and medical history questionnaire. The length of education was stated in predefined categories. Smoking was reported as never, former, and current smoker and the number of cigarettes, cigars, cheroots, and tobacco pipes smoked per day. Current tobacco consumption was calculated in grams per day by summing up the reported numbers per day with the use of conversion factors of 1.0 for cigarettes, 4.5 for cigars, and 3.0 for cheroots and tobacco pipes. Leisure time physical activity during the past year was assessed on the basis of questions about the mean number of hours per week spent on six types of activities (walking, biking, housework, home maintenance work, gardening and sports). Leisure time physical activity was defined as time spent on biking and sports. Hypertension was defined as reporting a history of hypertension or indicating use of anti-hypertensive medication. Diabetes mellitus was defined as a history of diabetes obtained through the Danish National Diabetes Register, reporting a history of diabetes or use of insulin. Information on alcohol consumption was obtained from the semi-quantitative food frequency questionnaire and reported as the mean amount of alcohol consumed during the previous year.

Height (without shoes) was measured to the nearest 0.5 cm, and weight was measured to the nearest 0.1 kg with the use of a digital scale and participants wearing light underwear. Body mass index is calculated as weight in kilograms divided by the square of height in meters.

### Statistical analysis

The characteristics of the subcohort participants and the cases are described by medians and 80% central ranges or by percentages.

Hazard ratios with 95% confidence intervals were used to describe the associations between the exposure total 18:1t, 18:1 Δ6-10t, 18:1 Δ11t and 18:2 Δ9c, 11t and the rate of MI. Hazard ratios were calculated using Cox proportional hazards regression with age as the time axis. To take into account the two-stage case-cohort design, we used the weighting method described by Kalbfleisch & Lawless [19] with robust variance estimators. Analyses were carried out separately for women and men because of the differences in the baseline hazards of MI. The exposure variables were included as continuous variables using restricted cubic spline regression with four knots. Splines were plotted to visually assess the shape of the associations with the 10 percentile as reference (i.e., log hazard ratio, 0.00 for the 10 percentile), and spline curves were formally tested against a horizontal line using Wald tests. Furthermore, hazard ratios were reported at the 30, 50, 70 and 90 percentiles with the 10 percentile as reference (i.e., hazard ratio, 1.00 for the 10 percentile). Tests for trend were calculated by including the exposure variables as continuous variables in the regression models. The covariates were as follows: Age at entry into the study, body mass index (kg/m^2^), length of education (≤7, 8–10 and >10 years), smoking (never, former and current smoker <15, 15–24 or ≥25 g tobacco/day), leisure time physical activity (≤3.5 and >3.5 hours per week), alcohol consumption (g/day), history of hypertension (yes, no, unknown) and history of diabetes mellitus (yes, no, unknown). The exposures 18:1 Δ6-10t, 18:1 Δ11t and 18:2 Δ9c, 11t were included simultaneously (i.e., mutually adjusted) in analyses of these exposures and MI. This was done in an attempt to isolate the associations for the individual TFAs. Adjustment for continuous variables was made using restricted cubic spline regression with three knots. The proportional hazards assumption was visually assessed with log-minus-log plots.

Data analyses were performed using Stata statistical software, release 13.1 (Stata Corporation, College Station, TX, USA).

## Results

After excluding participants with a cancer diagnosis not yet registered in the Danish Cancer Registry at the time of invitation (n = 564), participants with a diagnosis of MI or cardiac arrest prior to recruitment (n = 900) and participants who had not fully completed the questionnaires (n = 42), a total of 2376 cases were identified and 3409 participants remained in the subcohort (S1 Fig). Further, excluding participants with no information on exposures (n = 387) or covariates (n = 73) left 3156 participants in the subcohort and 2148 cases for study (S1 Fig). Characteristics of the subcohort participants and cases are shown in Table 1. The median follow-up time was 14 years (80% central range being from 13 to15).

**Table 1:** Characteristics of the subcohort participants and cases at enrolment: Diet, Cancer and Health cohort, Denmark.

	Women		Men	
	Subcohort	Cases	Subcohort	Cases
	n = 1469*	n = 584	n = 1687^†^	n = 1564
Sociodemographic				
Age (y)	56 (51–63)^‡^	60 (53–64)	56 (51–63)	58 (52–64)
Postmenopausal (%)	58	71		
Length of education, ≤7 years (%)	31	44	34	43
Body mass index (kg/m^2^)	25 (21–31)	26 (21–34)	26 (23–31)	27 (23–32)
Lifestyle				
Current smoker (%)	33	53	38	52
Leisure time physical activity, ≤3.5 hours/week (%)	65	71	66	69
Alcohol consumption (g/d)	9.5 (1.2–34.8)	6.4 (0.5–32.3)	19.7 (3.3–62.2)	18.2 (2.3–63.0)
Content of adipose tissue TFAs (% of total fatty acids)				
Total 18:1t	1.42 (1.01–1.98)	1.47 (1.05–2.06)	1.46 (1.03–1.97)	1.49 (1.02–2.03)
18:1 Δ6-10t	1.14 (0.80–1.66)	1.19 (0.81–1.75)	1.18 (0.81–1.65)	1.20 (0.82–1.69)
18:1 Δ11t	0.26 (0.17–0.38)	0.26 (0.17–0.35)	0.27 (0.18–0.40)	0.28 (0.17–0.39)
18:2 Δ9c, 11t	0.45 (0.34–0.58)	0.44 (0.33–0.59)	0.42 (0.31–0.53)	0.41 (0.31–0.53)
Medical history				
Hypertension (%)	17	33	15	23
Diabetes (%)	1	4	3	5

TFAs, indicates trans fatty acids.

*Including 32 myocardial infarction cases.

^†^Including 101 myocardial infarction cases.

^‡^Median (80% central range).

### Hazard ratio during 14 years of follow-up for the association between adipose tissue content of TFAs and MI

**Fig 1:**
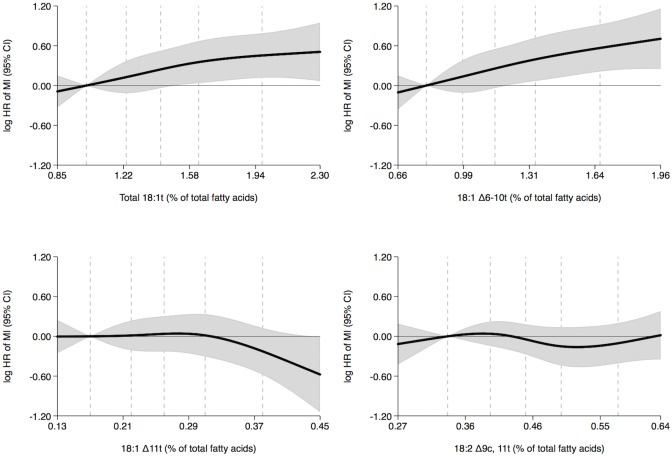
Adipose tissue trans fatty acid content (% of total fatty acids) and MI rate in women during 14 years of follow-up with the 10 percentile trans fatty acid content as reference: Diet, Cancer and Health cohort, Denmark. Shaded gray areas show 95% confidence intervals of HRs of MI (curves) that were calculated with the use of Cox proportional hazards regression and adjusted for age at entry into the study, body mass index, length of education, smoking status, leisure time physical activity level, alcohol consumption and history of hypertension and diabetes mellitus. The exposures 18:1 Δ6-10t, 18:1 Δ11t and 18:2 Δ9c, 11t were mutually adjusted. Analyses included 584 cases. Curves were formally tested against a horizontal line using Wald tests. The P-values were 0.070, 0.015, 0.186 and 0.516 in analyses of total 18:1t, 18:1 Δ6-10t, 18:1 Δ11t and 18:2 Δ9c, 11t, respectively. The 10, 30, 50, 70 and 90 percentiles of adipose tissue trans fatty acid content are marked by dashed lines. MI indicates myocardial infarction and HR, hazard ratio.

**Fig 2:**
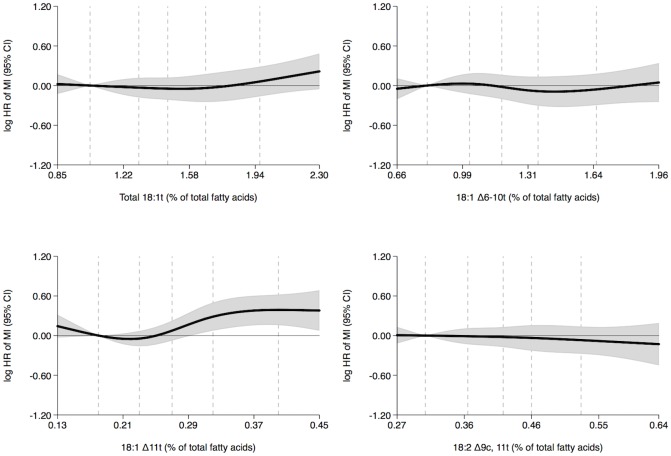
Adipose tissue trans fatty acid content (% of total fatty acids) and MI rate in men during 14 years of follow-up with the 10 percentile trans fatty acid content as reference: Diet, Cancer and Health cohort, Denmark. Shaded gray areas show 95% confidence intervals of HRs of MI (curves) that were calculated with the use of Cox proportional hazards regression and adjusted for age at entry into the study, body mass index, length of education, smoking status, leisure time physical activity level, alcohol consumption and history of hypertension and diabetes mellitus. The exposures 18:1 Δ6-10t, 18:1 Δ11t and 18:2 Δ9c, 11t were mutually adjusted. Analyses included 1564 cases. Curves were formally tested against a horizontal line using Wald tests. The P-values were 0.198, 0.595, 0.002 and 0.877 in analyses of total 18:1t, 18:1 Δ6-10t, 18:1 Δ11t and 18:2 Δ9c, 11t, respectively. The 10, 30, 50, 70 and 90 percentiles of adipose tissue trans fatty acid content are marked by dashed lines. MI indicates myocardial infarction and HR, hazard ratio.

In women, we observed positive trends between the adipose tissue content of total 18:1t and 18:1 Δ6-10t and the rate of MI (Fig 1 and Table 2). Women with higher total 18:1t content had a 57% higher MI rate (quintiles 5 versus 1, hazard ratio, 1.57; 95% confidence interval, 1.12–2.20; P-trend = 0.011), and women with higher 18:1 Δ6-10t content had a 76% higher MI rate (quintiles 5 versus 1, hazard ratio, 1.76; 95% confidence interval, 1.23–2.51; P-trend = 0.002) (Table 2). No association between adipose tissue 18:1 Δ11t content and MI rate was observed (Fig 1 and Table 2). In men, no associations between the adipose tissue content of total 18:1t and 18:1 Δ6-10t and the rate of MI were observed (Fig 2 and Table 2). However, a positive trend between adipose tissue 18:1 Δ11t content and MI rate was observed (Fig 2 and Table 2). Men with higher 18:1 Δ11t content had a 48% higher MI rate (quintiles 5 versus 1, hazard ratio, 1.48; 95% confidence interval, 1.17–1.86; P-trend = 0.003) (Table 2). The adipose tissue 18:2 Δ9c, 11t content was not associated with the MI rate in women or men (Figs 1 and 2 and Table 2).

**Table 2:** HR during 14 years of follow-up and 95% CI for the association between quintiles of adipose tissue content of trans fatty acids and myocardial infarction: Diet, Cancer and Health cohort, Denmark.

		Women (584 cases)				Men (1564 cases)			
			Model 1*	Model 2^†^	P-trend		Model 1*	Model 2^†^	P-trend
	Percentile	% of total fatty acids	HR (95% CI)	HR (95% CI)		% of total fatty acids	HR (95% CI)	HR 95% CI	
Total 18:1t	10	1.01	1.00	1.00	0.011	1.03	1.00	1.00	0.175
	30	1.23	1.00 (0.81–1.24)	1.13 (0.89–1.44)		1.30	0.89 (0.78–1.01)	0.97 (0.84–1.12)	
	50	1.42	1.04 (0.82–1.33)	1.27 (0.96–1.69)		1.46	0.88 (0.76–1.01)	0.95 (0.81–1.13)	
	70	1.63	1.11 (0.86–1.44)	1.42 (1.04–1.95)		1.67	0.91 (0.76–1.09)	0.96 (0.78–1.19)	
	90	1.98	1.20 (0.92–1.56)	1.57 (1.12–2.20)		1.97	1.00 (0.83–1.20)	1.06 (0.85–1.33)	
18:1 Δ6-10t	10	0.80	1.00	1.00	0.002	0.81	1.00	1.00	0.937
	30	0.98	1.07 (0.86–1.33)	1.14 (0.90–1.46)		1.02	0.98 (0.85–1.12)	1.03 (0.88–1.20)	
	50	1.14	1.12 (0.87–1.44)	1.29 (0.96–1.73)		1.18	0.96 (0.83–1.12)	0.98 (0.82–1.18)	
	70	1.34	1.18 (0.91–1.53)	1.48 (1.07–2.05)		1.36	0.96 (0.81–1.15)	0.92 (0.74–1.14)	
	90	1.66	1.31 (1.00–1.71)	1.76 (1.23–2.51)		1.65	1.03 (0.85–1.24)	0.94 (0.74–1.20)	
18:1 Δ11t	10	0.17	1.00	1.00	0.101	0.18	1.00	1.00	0.003
	30	0.22	0.97 (0.80–1.18)	1.01 (0.81–1.27)		0.23	0.90 (0.81–1.00)	0.95 (0.85–1.07)	
	50	0.26	1.00 (0.79–1.25)	1.04 (0.80–1.35)		0.27	0.92 (0.81–1.05)	1.08 (0.93–1.25)	
	70	0.31	0.98 (0.76–1.28)	1.02 (0.74–1.40)		0.32	0.99 (0.82–1.18)	1.33 (1.08–1.65)	
	90	0.38	0.74 (0.56–0.99)	0.80 (0.56–1.13)		0.40	0.97 (0.80–1.16)	1.48 (1.17–1.86)	
18:2 Δ9c, 11t	10	0.34	1.00	1.00	0.883	0.31	1.00	1.00	0.433
	30	0.40	0.95 (0.82–1.11)	1.04 (0.87–1.25)		0.37	0.97 (0.86–1.08)	0.99 (0.88–1.12)	
	50	0.45	0.84 (0.69–1.01)	0.96 (0.76–1.20)		0.42	0.93 (0.81–1.07)	0.98 (0.84–1.14)	
	70	0.50	0.74 (0.58–0.94)	0.86 (0.64–1.15)		0.46	0.91 (0.77–1.09)	0.96 (0.80–1.17)	
	90	0.58	0.76 (0.58–0.98)	0.90 (0.67–1.22)		0.53	0.89 (0.74–1.07)	0.93 (0.76–1.15)	

HR indicates hazard ratio and CI, confidence interval.

*HRs were calculated using Cox proportional hazards regression and adjusted for age at entry into the study.

^†^As model 1 plus body mass index, length of education, smoking status, leisure time physical activity level, alcohol consumption and history of hypertension and diabetes mellitus. The exposures 18:1 Δ6-10t, 18:1 Δ11t and 18:2 Δ9c, 11t were mutually adjusted. Tests for trend were calculated by including the exposure variables as continuous variables in the regression models.

### Hazard ratio during five years of follow-up for the association between adipose tissue content of TFAs and MI

**Fig 3:**
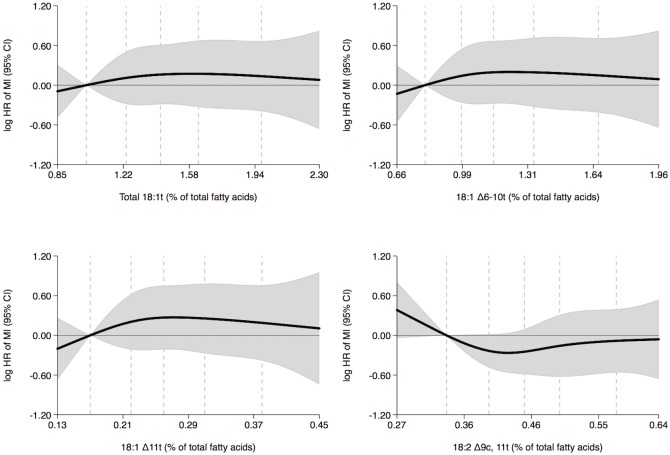
Adipose tissue trans fatty acid content (% of total fatty acids) and MI rate in women during five years of follow-up with the 10 percentile trans fatty acid content as reference: Diet, Cancer and Health cohort, Denmark. Shaded gray areas show 95% confidence intervals of HRs of MI (curves) that were calculated with the use of Cox proportional hazards regression and adjusted for age at entry into the study, body mass index, length of education, smoking status, leisure time physical activity level, alcohol consumption and history of hypertension and diabetes mellitus. The exposures 18:1 Δ6-10t, 18:1 Δ11t and 18:2 Δ9c, 11t were mutually adjusted. Analyses included 163 cases. Curves were formally tested against a horizontal line using Wald tests. The P-values were 0.915, 0.874, 0.742 and 0.331 in analyses of total 18:1t, 18:1 Δ6-10t, 18:1 Δ11t and 18:2 Δ9c, 11t, respectively. The 10, 30, 50, 70 and 90 percentiles of adipose tissue trans fatty acid content are marked by dashed lines. MI indicates myocardial infarction and HR, hazard ratio.

**Fig 4:**
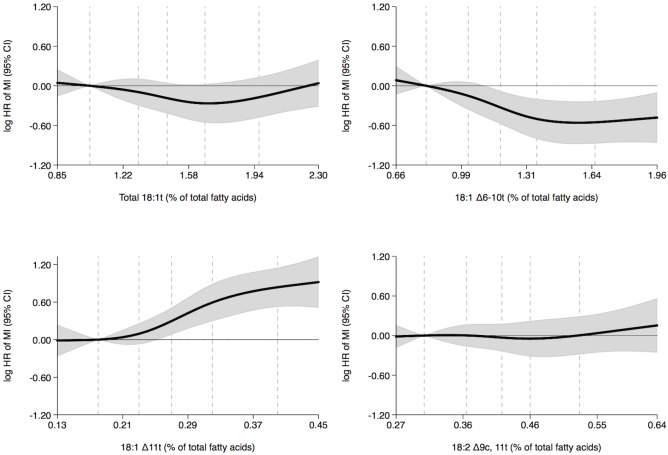
Adipose tissue trans fatty acid content (% of total fatty acids) and MI rate in men during five years of follow-up with the 10 percentile trans fatty acid content as reference: Diet, Cancer and Health cohort, Denmark. Shaded gray areas show 95% confidence intervals of HRs of MI (curves) that were calculated with the use of Cox proportional hazards regression and adjusted for age at entry into the study, body mass index, length of education, smoking status, leisure time physical activity level, alcohol consumption and history of hypertension and diabetes mellitus. The exposures 18:1 Δ6-10t, 18:1 Δ11t and 18:2 Δ9c, 11t were mutually adjusted. Analyses included 547 cases. Curves were formally tested against a horizontal line using Wald tests. The P-values were 0.150, 0.009, <0.001 and 0.834 in analyses of total 18:1t, 18:1 Δ6-10t, 18:1 Δ11t and 18:2 Δ9c, 11t, respectively. The 10, 30, 50, 70 and 90 percentiles of adipose tissue trans fatty acid content are marked by dashed lines. MI indicates myocardial infarction and HR, hazard ratio.

In women, no associations between the adipose tissue content of TFAs and the rate of MI were observed (Fig 3 and Table 3). In men, no associations between the adipose tissue content of total 18:1t and the rate of MI was observed (Fig 4 and Table 3). However, a negative trend between adipose tissue 18:1 Δ6-10t content and MI rate and a positive trend between adipose tissue 18:1 Δ11t content and MI rate were observed (Fig 4 and Table 3). Men with higher 18:1 Δ6-10t content had a 43% lower MI rate (quintiles 5 versus 1, hazard ratio, 0.57; 95% confidence interval, 0.42–0.79; P-trend = 0.014) and men with higher 18:1 Δ11t content had a 131% higher MI rate (quintiles 5 versus 1, hazard ratio, 2.31; 95% confidence interval, 1.69–3.16; P-trend = <0.001) (Table 3). Adipose tissue 18:2 Δ9c, 11t content was not associated with MI rate in men (Fig 4 and Table 3).

**Table 3:** HR during five years of follow-up and 95% CI for the association between quintiles of adipose tissue content of trans fatty acids and myocardial infarction: Diet, Cancer and Health cohort, Denmark.

		Women (163 cases)				Men (547 cases)			
			Model 1*	Model 2^†^	P-trend		Model 1*	Model 2^†^	P-trend
	Percentile	% of total fatty acids	HR (95% CI)	HR (95% CI)		% of total fatty acids	HR (95% CI)	HR (95% CI)	
Total 18:1t	10	1.01	1.00	1.00	0.759	1.03	1.00	1.00	0.999
	30	1.23	1.02 (0.70–1.49)	1.12 (0.75–1.66)		1.30	0.88 (0.73–1.06)	0.91 (0.74–1.11)	
	50	1.42	0.99 (0.65–1.51)	1.17 (0.75–1.84)		1.46	0.83 (0.68–1.02)	0.83 (0.66–1.05)	
	70	1.63	0.95 (0.61–1.47)	1.19 (0.72–1.96)		1.67	0.82 (0.63–1.05)	0.77 (0.57–1.03)	
	90	1.98	0.90 (0.57–1.41)	1.15 (0.67–1.95)		1.97	0.90 (0.69–1.16)	0.84 (0.62–1.14)	
18:1 Δ6-10t	10	0.80	1.00	1.00	0.867	0.81	1.00	1.00	0.014
	30	0.98	1.16 (0.79–1.72)	1.15 (0.77–1.73)		1.02	0.96 (0.80–1.16)	0.86 (0.70–1.06)	
	50	1.14	1.15 (0.73–1.79)	1.21 (0.76–1.95)		1.18	0.90 (0.72–1.11)	0.72 (0.56–0.93)	
	70	1.34	1.03 (0.66–1.61)	1.21 (0.72–2.05)		1.36	0.84 (0.65–1.08)	0.61 (0.45–0.82)	
	90	1.66	0.97 (0.61–1.54)	1.16 (0.66–2.04)		1.65	0.87 (0.67–1.13)	0.57 (0.42–0.79)	
18:1 Δ11t	10	0.17	1.00	1.00	0.623	0.18	1.00	1.00	<0.001
	30	0.22	1.07 (0.73–1.58)	1.23 (0.81–1.87)		0.23	0.97 (0.83–1.13)	1.10 (0.93–1.30)	
	50	0.26	1.08 (0.70–1.66)	1.31 (0.81–2.12)		0.27	1.02 (0.84–1.24)	1.35 (1.08–1.67)	
	70	0.31	0.99 (0.64–1.54)	1.29 (0.76–2.19)		0.32	1.14 (0.88–1.48)	1.81 (1.34–2.44)	
	90	0.38	0.78 (0.49–1.24)	1.21 (0.68–2.13)		0.40	1.27 (0.98–1.63)	2.31 (1.69–3.16)	
18:2 Δ9c, 11t	10	0.34	1.00	1.00	0.560	0.31	1.00	1.00	0.688
	30	0.40	0.73 (0.60–0.89)	0.79 (0.61–1.02)		0.37	0.97 (0.83–1.13)	1.00 (0.85–1.19)	
	50	0.45	0.67 (0.52–0.88)	0.78 (0.56–1.10)		0.42	0.92 (0.76–1.11)	0.97 (0.79–1.20)	
	70	0.50	0.69 (0.47–1.03)	0.85 (0.53–1.36)		0.46	0.89 (0.70–1.14)	0.95 (0.73–1.25)	
	90	0.58	0.72 (0.47–1.09)	0.92 (0.57–1.49)		0.53	0.96 (0.74–1.24)	1.00 (0.75–1.34)	

HR indicates hazard ratio and CI, confidence interval.

*HRs were calculated using Cox proportional hazards regression and adjusted for age at entry into the study.

^†^As model 1 plus body mass index, length of education, smoking status, leisure time physical activity level, alcohol consumption and history of hypertension and diabetes mellitus. The exposures 18:1 Δ6-10t, 18:1 Δ11t and 18:2 Δ9c, 11t were mutually adjusted. Tests for trend were calculated by including the exposure variables as continuous variables in the regression models.

## Discussion

Among women, a positive trend between the adipose tissue content of 18:1 Δ6-10t and the rate of MI was observed during 14 years of follow-up, whereas no association between adipose tissue 18:1 Δ11t content and MI rate was observed. In contrast, among men, no association between the adipose tissue content of 18:1 Δ6-10t and the rate of MI was observed during 14 years of follow-up, but a positive trend between adipose tissue 18:1 Δ11t content and MI rate was seen. The adipose tissue 18:2 Δ9c, 11t content was not associated with the MI rate in women or men.

Analyses were conducted separately among women and men because of the differences in the baseline hazards of MI. Men have a higher baseline hazard of MI than women. Consequently, associations on a relative scale were expected to be weaker among men than among women. Surprisingly, we observed a positive association between adipose tissue 18:1 Δ6-10t content and MI rate among women but not among men and a positive association between adipose tissue 18:1 Δ11t content and MI rate among men but not among women. There are no other epidemiological studies with relevant results and there is a lack of evidence to suggest biological mechanisms underlying differential effects. More research on the subject is needed.

**Fig 5:**
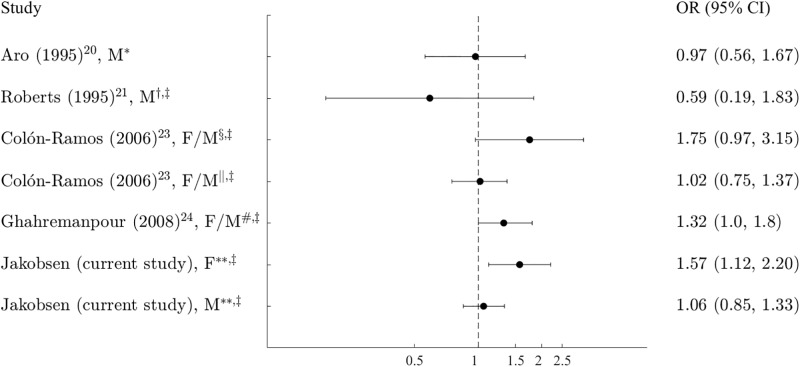
Population-based case-control studies on total 18:1t in adipose tissue and odds of myocardial infarction. *Highest versus lowest quartile of total 18:1t. †OR of sudden cardiac death. ‡Highest versus lowest quintile of total 18:1t. §Before a reduction of trans fatty acids in foods. ||After a reduction of trans fatty acids in foods. #OR of coronary artery stenosis. **Hazard ratio of myocardial infarction. OR indicates odds ratio; CI, confidence interval; M, male and F, female.

The association between the adipose tissue content of total 18:1t and the risk of coronary heart disease has been investigated in five case-control studies [20–24]. Among these studies, one study, including both women and men, observed a higher rate of coronary artery stenosis for a high adipose tissue content of total 18:1t [24], whereas two studies including only men reported no associations between total 18:1t and MI [20] or sudden cardiac death [21] (Fig 5). In women and men combined, Colón-Ramos et al. [23] observed a higher rate of MI for a high adipose tissue content of total 18:1t before a reduction of TFAs in foods but not after (Fig 5). Dlouhý et al. [22] found a higher average adipose tissue content of total 18:1t (2.31 ± 1.09%) in cardiac patients compared with the average content of total 18:1t (1.95 ± 0.77%) in non-cardiac patients (P-value 0.05). However, the results from most of these previous studies were hampered by small study sizes. We observed a positive association between adipose tissue content of total 18:1t and rate of MI in women but not in men. Clifton et al. [11] investigated the associations between the content of individual adipose tissue 18:1t isomers and the rate of MI among women and men. A higher rate of MI was found for a high content of 18:1 Δ11t, whereas no associations were found for the content of 18:1 Δ9t and 18:1 Δ10t. These findings are in line with our findings in men but in contrast to our findings in women. Although, the adipose tissue 18:1 Δ11t content was considerably lower in the subcohort participants in our study (80% central range being from 0.17% to 0.38% of fatty acids among women and 0.18% to 0.40% of fatty acids among men) compared with the adipose tissue 18:1 Δ11t content in the population-based controls in the study by Clifton et al. [11] (mean ± SD being 0.54 ± 0.21 g/100 g fatty acids), the lower 18:1 Δ11t content does not explain the finding of no association among women in our study, as the 18:1 Δ11t content was similar among women and men. Smit et al. [12] investigated the association between the content of adipose tissue 18:2 Δ9c, 11t and the rate of MI among women and men and found a negative association. In our study, no association was observed in women or men. An explanation for this might be the lower adipose tissue 18:2 Δ9c, 11t content in our population. In the study by Smit et al. [12], the median adipose tissue 18:2 Δ9c, 11t content ranged from 0.35% of total fatty acids in the lowest quintile to 0.78% of total fatty acids in the highest quintile among the population-based controls. In our study, the 80% central range was 0.34% to 0.58% of total fatty acids among women in the subcohort and 0.31% to 0.53% of total fatty acids among men in the subcohort.

In supplementary analyses, the study was halted after five years of follow-up. In women, no association between the adipose content of 18:1 Δ6-10t and the rate of MI was observed during the five years of follow-up in contrast to the positive trend between the adipose 18:1 Δ6-10t content and the MI rate observed during the 14 years of follow-up. This finding was surprising as we would have expected the associations between TFAs and MI rate to be weaker when the study was halted after 14 years of follow-up due to the aging of the population and due to the presumed declining exposure to industrial TFAs during follow-up. During the 1990s, the content of TFAs in Danish margarines declined, and in 1999, TFAs were practically absent from Danish margarines [25]. Furthermore, in 2003, Denmark imposed a regulation specifying a maximum of 2 g of industrially produced TFAs per 100 g of oil or fat intended for human consumption. Thus, the exposure to industrial TFAs has probably declined during follow-up. This is also supported by national surveys reporting declines in intake of industrial TFAs during the 2000s [26]. In men, a negative trend between the adipose tissue content of 18:1 Δ6-10t and the rate of MI was observed during the five years of follow-up; this was not evident after 14 years of follow-up. Furthermore, a stronger positive trend between the adipose tissue 18:1 Δ11t content and the MI rate was observed during the five years of follow-up compared with the association observed during the 14 years of follow-up. The adipose tissue 18:2 Δ9c, 11t content was not associated with the MI rate in women or men during 5 or 14 years of follow-up.

Some strengths and limitations of the current study should be mentioned. All residents aged 50–64 years without a diagnosis of cancer registered in the Danish Cancer Registry and living in two defined urban and suburban areas of Denmark were invited to participate in the Diet, Cancer and Health cohort. The fact that the participants who agreed to participate were socioeconomically more affluent than those who declined the invitation [13] may influence generalization of the results to persons socioeconomically less affluent. We do, however, expect the direction of the associations between the adipose tissue content of TFAs and the rate of MI to be independent of socioeconomic status. Loss to follow-up was solely due to emigration, and only 0.5% emigrated during follow-up, rendering selection bias due to attrition unlikely. Information on cases of MI was validated. Furthermore, information bias is unlikely to have affected the results as information on cases of MI was obtained by linkage with registers, and thus case ascertainment was independent of TFA measurement. A major strength of the study is the use of adipose tissue content of TFAs as a long-term biomarker of TFA intake and metabolism [10]. Moreover, adipose tissue content of TFAs is an objective measure minimizing random measurement error. Also, we attempted to address confounding by careful adjustment for established risk factors of coronary heart disease. However, residual confounding is possible. Furthermore, in an attempt to isolate the associations for the individual TFAs, we performed mutual adjustment. Mutual adjustment for TFAs, however, complicates interpretation of the results as restrictions in the underlying food sources are introduced; thereby probably referring to different underlying dietary patterns.

In conclusion, in this study the content of 18:2 Δ9c, 11t in adipose tissue was not associated with the rate of MI in women or men, whereas higher contents of isomers of 18:1t were associated with higher rates of MI but the associations for individual 18:1t isomers differed, however, in women and men.

## Supporting information

S1 Fig Flowchart for the selection of subcohort participants and cases: Diet, Cancer and Health cohort, Denmark. MI indicates myocardial infarction.
(PDF)

## References

[1] Precht D, Molkentin J. Trans fatty acids: Implications for health, analytical methods, incidence in edible fats and intake (a review). Nahrung. 1995;39:343–374. 8569844 10.1002/food.19950390503

[2] Precht D, Molkentin J. Rapid analysis of the isomers of trans-octadecenoic acid in milk fat. Int Dairy J. 1996;6:791–809.

[3] Turpeinen AM, Mutanen M, Aro A, Salminen I, Basu S, Palmquist DL et al Bioconversion of vaccenic acid to conjugated linoleic acid in humans. Am J Clin Nutr. 2002;76:504–510. 10.1093/ajcn/76.3.504 12197992

[4] Jakobsen MU, Bysted A, Andersen NL, Heitmann BL, Hartkopp HB, Leth K et al Intake of ruminant trans fatty acids and risk of coronary heart disease–an overview. Atheroscler Suppl. 2006;7:9–11. 10.1016/j.atherosclerosissup.2006.04.004 16713389

[5] Jakobsen MU, Overvad K. Observational epidemiological studies on intake of trans fatty acids and risk of ischaemic heart disease In: Destaillats F, Sébédio JL, Fabiola D, Chardigny JM, editors. Trans fatty acids in human nutrition. Bridgwater, England: The Oily Press; 2009 pp. 255–306.

[6] Gebauer SK, Chardigny JM, Jakobsen MU, Lamarche B, Lock AL, Proctor SD et al Effects of ruminant trans fatty acids on cardiovascular disease and cancer: a comprehensive review of epidemiological, clinical, and mechanistic studies. Adv Nutr. 2011;2:332–354. 10.3945/an.111.000521 22332075 PMC3125683

[7] Bendsen NT, Christensen R, Bartels EM, Astrup A. Consumption of industrial and ruminant trans fatty acids and risk of coronary heart disease: a systematic review and meta-analysis of cohort studies. Eur J Clin Nutr. 2011;65:773–783. 10.1038/ejcn.2011.34 21427742

[8] Brouwer IA, Wanders AJ, Katan MB. Trans fatty acids and cardiovascular health: research completed? Eur J Clin Nutr. 2013;67:541–547. 10.1038/ejcn.2013.43 23531781

[9] Wang DD, Hu FB. Dietary fat and risk of cardiovascular disease: Recent controversies and advances. Annu Rev Nutr. 2017;37:423–446. 10.1146/annurev-nutr-071816-064614 28645222

[10] Hodson L, Skeaff CM, Fielding BA. Fatty acid composition of adipose tissue and blood in humans and its use as a biomarker of dietary intake. Prog Lipid Res. 2008;47:348–380. 10.1016/j.plipres.2008.03.003 18435934

[11] Clifton PM, Keogh JB, Noakes M. Trans fatty acids in adipose tissue and the food supply are associated with myocardial infarction. J Nutr. 2004;134:874–879. 10.1093/jn/134.4.874 15051840

[12] Smit LA, Baylin A, Campos H. Conjugated linoleic acid in adipose tissue and risk of myocardial infarction. Am J Clin Nutr. 2010;92:34–40. 10.3945/ajcn.2010.29524 20463040 PMC2884320

[13] Tjønneland A, Olsen A, Boll K, Stripp C, Christensen J, Engholm G et al Study design, exposure variables, and socioeconomic determinants of participation in Diet, Cancer and Health: a population-based prospective cohort study of 57,053 men and women in Denmark. Scand J Public Health. 2007;35:432–441. 10.1080/14034940601047986 17786808

[14] Beynen AC, Katan MB. Rapid sampling and long-term storage of subcutaneous adipose-tissue biopsies for determination of fatty acid composition. Am J Clin Nutr. 1985;42:317–322. 10.1093/ajcn/42.2.317 4025201

[15] Barlow WE, Ichikawa L, Rosner D, Izumi S. Analysis of case-cohort designs. J Clin Epidemiol. 1999;52:1165–1172. 10580779 10.1016/s0895-4356(99)00102-x

[16] Joensen AM, Overvad K, Dethlefsen C, Johnsen SP, Tjønneland A, Rasmussen LH et al Marine n-3 polyunsaturated fatty acids in adipose tissue and the risk of acute coronary syndrome. Circulation. 2011;124:1232–1238. 10.1161/CIRCULATIONAHA.110.987057 21859970

[17] Joensen AM, Jensen MK, Overvad K, Dethlefsen C, Schmidt E, Rasmussen L et al Predictive values of acute coronary syndrome discharge diagnoses differed in the Danish National Patient Registry. J Clin Epidemiol. 2009;62:188–194. 10.1016/j.jclinepi.2008.03.005 18722087

[18] Stegger JG, Schmidt EB, Obel T, Berentzen TL, Tjønneland A, Sørensen TIA et al Body composition and body fat distribution in relation to later risk of acute myocardial infarction: a Danish follow-up study. Int J Obes. 2011;35:1433–1441. 10.1038/ijo.2010.278 21285940

[19] Kalbfleisch JD, Lawless JF. Likelihood analysis of multi-state models for disease incidence and mortality. Stat Med. 1988;7:149–160. 3353602 10.1002/sim.4780070116

[20] Aro A, Kardinaal AF, Salminen I, Kark JD, Riemersma RA, Delgado-Rodriguez M et al Adipose tissue isomeric trans fatty acids and risk of myocardial infarction in nine countries: the EURAMIC study. Lancet. 1995;345:273–278. 7766242 10.1016/s0140-6736(95)90273-2

[21] Roberts TL, Wood DA, Riemersma RA, Gallagher PJ, Lampe FC. Trans isomers of oleic and linoleic acids in adipose tissue and sudden cardiac death. Lancet. 1995;345:278–282. 7837861 10.1016/s0140-6736(95)90274-0

[22] Dlouhý P, Tvrzická E, Stanková B, Vecka M, Žák A, Straka Z et al Higher content of 18:1 trans fatty acids in subcutaneous fat of persons with coronarographically documented atherosclerosis of the coronary arteries. Ann Nutr Metab. 2003;47:302–305. 10.1159/000072403 14520026

[23] Colón-Ramos U, Baylin A, Campos H. The relation between trans fatty acid levels and increased risk of myocardial infarction does not hold at lower levels of trans fatty acids in the Costa Rican food supply. J Nutr. 2006;136:2887–2892. 10.1093/jn/136.11.2887 17056818

[24] Ghahremanpour F, Firoozrai M, Darabi M, Zavarei A, Mohebbi A. Adipose tissue trans fatty acids and risk of coronary artery disease: a case-control study. Ann Nutr Metab. 2008;52:24–28. 10.1159/000114291 18230967

[25] Leth T, Bysted A, Hansen K, Ovesen L. Trans FA content in Danish margarines and shortenings. J Am Oil Chem Soc. 2003;80:475–478.

[26] Wanders AJ, Zock PL, Brouwer IA. Trans fat intake and its dietary sources in general populations worldwide: A systematic review. Nutrients. 2017;9. pii: E840. 10.3390/nu9080840 PMC5579633 28783062

