# Supplementary material for: Trans fatty acids in adipose tissue and risk of myocardial infarction: A case-cohort study
Source: PLoS One. 2018 Aug 22;13(8):e0202363. doi: 10.1371/journal.pone.0202363 (PMC6104995; doi:10.1371/journal.pone.0202363)
Supplement: S1 Fig — MI indicates myocardial infarction. (PDF) [file pone.0202363.s001.pdf]

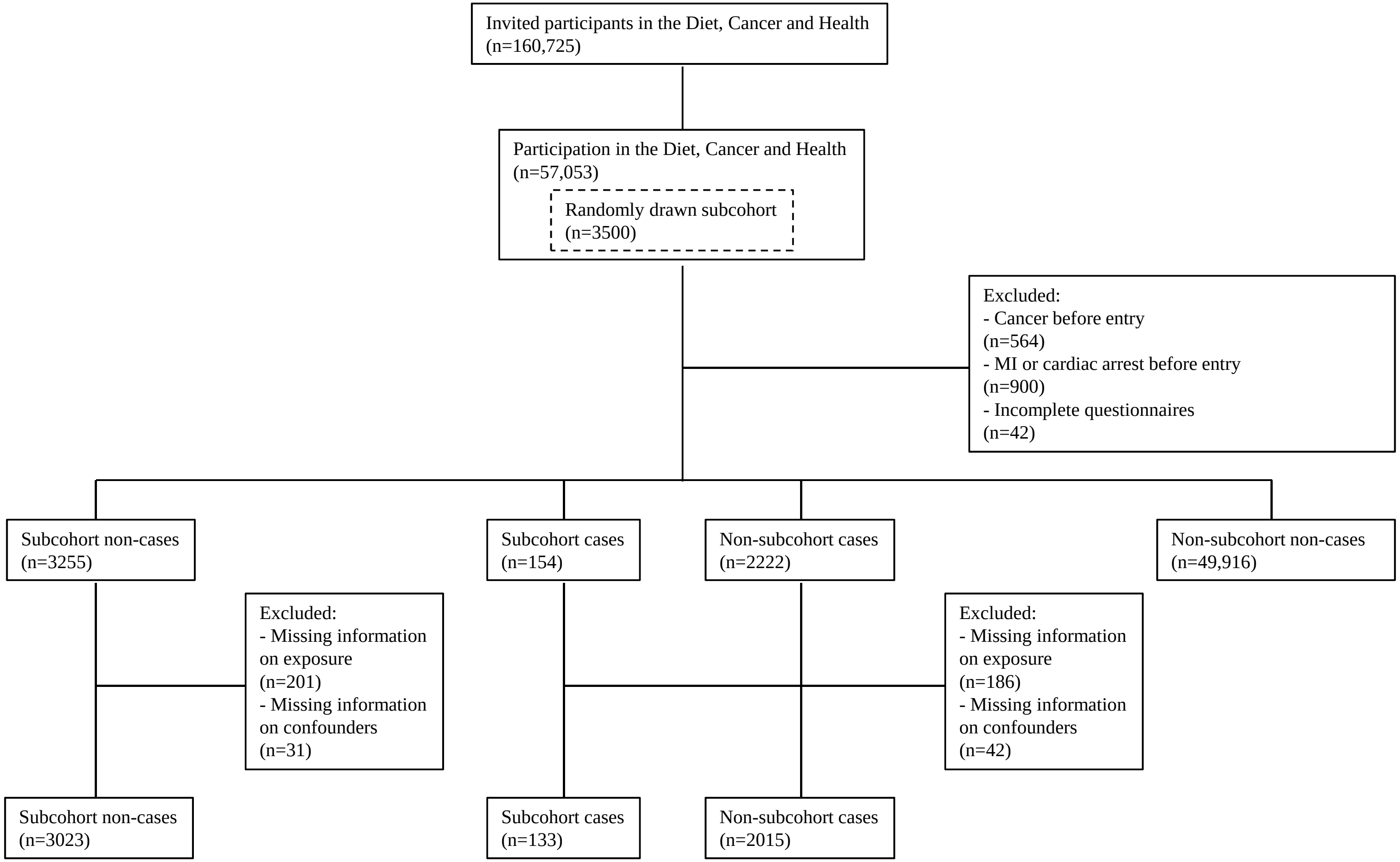

S1 Fig. Flowchart for the selection of subcohort participants and cases: Diet, Cancer and Health cohort, Denmark. MI indicates myocardial infarction.
